# Investigating the Causes for Decreased Levels of Glutathione in Individuals with Type II Diabetes

**DOI:** 10.1371/journal.pone.0118436

**Published:** 2015-03-19

**Authors:** Minette Lagman, Judy Ly, Tommy Saing, Manpreet Kaur Singh, Enrique Vera Tudela, Devin Morris, Po-Ting Chi, Cesar Ochoa, Airani Sathananthan, Vishwanath Venketaraman

**Affiliations:** 1 Graduate College of Biomedical Sciences, Western University of Health Sciences, Pomona, California, United States of America; 2 Department of Basic Medical Sciences, College of Osteopathic Medicine of the Pacific, Western University of Health Sciences, Pomona, California, United States of America; 3 Western Diabetes Institute, Pomona, California, United States of America; Center for Cancer Research, National Cancer Institute, UNITED STATES

## Abstract

Tuberculosis (TB) remains an eminent global burden with one third of the world’s population latently infected with *Mycobacterium tuberculosis* (*M*. *tb*). Individuals with compromised immune systems are especially vulnerable to *M*. *tb* infection. In fact, individuals with Type 2 Diabetes Mellitus (T2DM) are two to three times more susceptible to TB than those without T2DM. In this study, we report that individuals with T2DM have lower levels of glutathione (GSH) due to compromised levels of GSH synthesis and metabolism enzymes. Transforming growth factor beta (TGF-β), a cytokine that is known to decrease the expression of the catalytic subunit of glutamine-cysteine ligase (GCLC) was found in increased levels in the plasma samples from individuals with T2DM, explaining the possible underlying mechanism that is responsible for decreased levels of GSH in individuals with T2DM. Moreover, increased levels of pro-inflammatory cytokines such as interleukin-6 (IL-6) and interleukin-17 (IL-17) were observed in plasma samples isolated from individuals with T2DM. Increased levels of IL-6 and IL-17 was accompanied by enhanced production of free radicals further indicating an alternative mechanism for the decreased levels of GSH in individuals with T2DM. Augmenting the levels of GSH in macrophages isolated from individuals with T2DM resulted in improved control of *M*. *tb* infection. Furthermore, cytokines that are responsible for controlling *M*. *tb* infection at the cellular and granuloma level such as tumor necrosis factor alpha (TNF-α), interleukin-1β (IL-1β), interleukin-2 (IL-2), interferon-gamma (IFN-γ), and interleukin-12 (IL-12), were found to be compromised in plasma samples isolated from individuals with T2DM. On the other hand, interleukin-10 (IL-10), an immunosuppressive cytokine was increased in plasma samples isolated from individuals with T2DM. Overall, these findings suggest that lower levels of GSH in individuals with T2DM lead to their increased susceptibility to *M*. *tb* infection.

## Introduction

As of August, 2014 one third of the world’s population remains latently infected with *Mycobacterium tuberculosis (M. tb)*, the causative agent for tuberculosis (TB) [WHO, 2014]. This number is a major cause for concern since TB is a leading cause of death especially in middle to low-income areas. In recent years, there has been a significant increase in the incidence of TB due to the emergence of multidrug and extensively-drug resistant strains of *M*. *tb*. Increased numbers of highly susceptible immunocompromised individuals such as those who are of old age, have a history of substance abuse, have HIV, or have diabetes mellitus, has also greatly contributed to increased cases of TB [1, 2].

The World Health Organization (WHO) reports that an estimated 347 million people all over the world have diabetes and expects the global prevalence to increase by up to 50% by year 2030 [3]. In the United States alone the Center for Disease Control (CDC) reports that 29.1 million people have diabetes, with approximately 1.9 million people diagnosed every year and a staggering 79 million Americans in pre-diabetic stage [4]. Type 2 diabetes mellitus (T2DM) is a condition characterized by hyperglycemia caused by insulin resistance. T2DM accounts for 90–95% of all diabetes prevalence [5].

Glycated hemoglobin (HbA1c) is representative of plasma glucose for eight to twelve weeks. The American Diabetes Association (ADA) and the International Expert Committee (IEC) established HbA1c as a diagnostic marker for T2DM. The HbA1c cut-off point for diagnosis of T2DM is set at 6.5% while HbA1c 5.7% to 6.4% is considered high risk for T2DM [6,7]. Individuals with T2DM are highly susceptible to bacterial infections [8,9]. In fact, patients with diabetes have two to three times higher risk of contracting TB compared to a healthy individual [10]. About 10% of TB cases are linked to diabetes. Individuals with diabetes also have a higher risk of death during TB treatment. They are also at a higher risk of TB relapse after treatment [1,3]. This risk is highly related to the chronic inflammation caused by marked increase in production of pro-inflammatory cytokines [11].

Cytokines are known to be regulated by GSH levels, as confirmed by a previous study conducted by our lab [12]. GSH is an antioxidant composed of glutamine, cysteine, and glycine. It is recognized for preventing cellular damage by detoxifying reactive oxygen species (ROS) [13]. GSH exist in two forms: reduced GSH (rGSH) and oxidized GSH (GSSG). rGSH has antioxidant function while GSSG is a byproduct of the oxidation of GSH and does not have antioxidant effects. In the presence of ROS, two molecules of rGSH are converted to the oxidized form, GSSG, and water [13].

The *de novo* synthesis of GSH involves two major steps: first, glutamine-cysteine ligase (GCL) links glutamine and cysteine to form γ–glutamylcysteine, then, glutathione synthetase (GSS) links γ–glutamylcysteine to glycine to form the final molecule of GSH. GCL is a heterodimer composed of a catalytic subunit (GCLC), which has an active site and catalytic activity responsible for linking glutamine and cysteine and a modulating subunit (GCLM), which enhances the effects of GCLC. TGF-β, a cytokine mainly produced by macrophages and regulatory T cells has suppressive effects on the functions of macrophages and T cells [12]. Studies have shown that TGF-β can also down-regulate the expression of GCLC ultimately leading to decreased levels of GSH [14,15].

GSH can also be synthesized by recycling GSSG back to GSH. This is achieved by glutathione reductase (GSR), which uses NADPH as a cofactor. GSR converts GSSG back to rGSH. The polyol pathway, where glucose is converted to fructose, has been implicated as one of the causes for decreased levels of GSH in individuals with T2DM. The first step in the polyol pathway is the conversion of glucose to sorbitol catalyzed by aldose reductase (AR) which also uses NADPH. AR competes with the GSR for NADPH. This competition leads to compromised levels of NADPH interfering with the GSH recycling pathway involving conversion of GSSG back to GSH and eventually resulting in diminished levels of GSH [11].

Another notable enzyme that plays an important role in the GSH metabolism is gamma glutamyl transpeptidase (GGT) [16]. Extracellular GSH cannot be transported as a whole molecule into the cell. GGT, which is expressed on the cell membrane cleaves GSH at the γ-glutamyl linkage to release glutamine and cysteinyl glycine [16,17]. Cysteinyl glycine is then cleaved by aminopeptidase to cysteine and glycine. Cystine is transported into the cells and is used as a reactant for the *de novo* synthesis of GSH [16,17].

In this study, we hypothesized that the levels of GSH are compromised in individuals with T2DM due to diminished levels of GSH synthesis and metabolism enzymes and this will impair the ability of macrophages to control *M*. *tb* infection. We also tested for other possible mechanisms that may be responsible for decreasing the levels of GSH in individuals with T2DM such as free radicals production in response to inflammatory cytokines (IL-6 and IL-17). Furthermore, we determined the levels of cytokines that are responsible for controlling *M*. *tb* infection in the plasma samples from individuals with T2DM and tested the effects of GSH-enhancing agents to improve the ability of monocyte-derived macrophages from individuals with T2DM to control *M*. *tb* infection. Our results unveil the causes for decreased levels of GSH in individuals with T2DM and establish the benefits of GSH-supplementation in improving the innate control *M*. *tb* infection.

## Materials and Methods

### Subjects

Twenty subjects (10 healthy individuals and 10 individuals with T2DM) were recruited through the Western Diabetes Institute, Western University of Health Sciences, after obtaining approvals from the Institutional Review Board (IRB) and the Institutional Biosafety Committee (IBC). All 20 participants were 25–65 years old and had healthy liver functions at the time of the study. All healthy participants had glycated hemoglobin (HbA1c) of less than 6 percent. Individuals with T2DM had HbA1c of 7–10% and were not taking metformin or glitazones. The purpose of the study and what was required from each participant was explained to each individual before obtaining their signed informed consent to be subjects in our study. After signing an informed consent form, thirty-five milliliters of blood were drawn from each participant in one visit.

### Quantification of GSH levels in plasma and cell lysates of monocytes and red blood cells (RBCs) from healthy individuals and individuals with T2DM

Measurement of total and oxidized glutathione was performed by the colorimetric method using an assay kit from Arbor Assays (K006-H1). Monocytes and plasma were first resuspended in cold 5% sulfosalicylic acid (SSA) dehydrate, and then centrifuged for 15 minutes at 13,000 rpm at 4°C. Supernatants collected after centrifugation were used for the GSH assay. RBCs were sonicated prior to resuspension in 5% SSA. GSH was assayed according to manufacturer’s instructions. Calculation of reduced GSH was achieved by subtracting GSSG from total GSH. All measurements were corrected for total protein levels and the results were reported in moles GSH per μg protein.

### Gel electrophoresis and western blot analysis of GSH synthesis enzymes in RBCs

Total protein concentration in the RBC lysates was determined by coomasie blue colorimetric assay. Ten micrograms (10 μg) of total RBC proteins per sample were separated by denaturing polyacrylamide electrophoresis with 12% polyacrylamide. Transfer of separated proteins was completed by electroblotting onto a polyvinylidene fluoride membrane. The membranes were blocked for one hour at room temperature with tris buffered saline containing tween 20 (TBST) and 5% nonfat dry milk, with mild shaking. The membranes were then incubated overnight with primary antibody diluted in blocking buffer at 4°C with mild shaking. Primary antibodies were GCLC (1:1000), GSS (1:1000), GSR (1:500), GGT (1:250), GAPDH (1:1000). Following incubation with primary antibody, the membranes were washed five times with TBST for 15 minutes at room temperature. The membranes were then incubated at room temperature for one hour with horseradish peroxidase-conjugated goat anti-rabbit or goat anti-mouse secondary antibody diluted with TBST followed by another set of washing with TBST. Chemiluminescent substrate was added onto the membrane followed by exposure to the X-ray film. Following this, the X-ray film was developed in the dark room. Resulting immunoblots were captured by Versadoc and densitometrically analyzed by Image J.

### Quantifying Reactive Oxygen Species (ROS) Levels in plasma and cell lysates from RBCs and monocytes, derived from healthy individuals and individuals with T2DM

ROS production was determined by two methods: In the first, cellROX staining of monocytes, CD4+ and CD8+ T cells was performed, followed by quantification of fluorescence by flow cytometry (FACS). In the second method, malondialdehyde or MDA (end product of lipid peroxidation-an indirect measure of ROS production) was measured in plasma samples and lysates of monocytes and RBCs from healthy subjects and individuals with T2DM by spectrophotometry.

### CellROX staining and flow cytometry analysis

Peripheral blood mononuclear cells (PBMCs) were isolated from whole blood of healthy subjects and individuals with T2DM by density centrifugation with 1:1 ratio of Ficoll-Paque PLUS (GE Healthcare, 10040757) at 1800 rpm. PBMCs from healthy subjects and individuals with T2DM were treated with 5 μM cellROX green reagent (Life Technologies, C10444) and incubated at room temperature for 30 minutes in dark. Stained PBMCs were centrifuged at 800 x g for 5 minutes and re-suspended in 100 μl PBS. Antibodies conjugated to the fluorescent markers such as CD14-PE (eBioscience, 12–0149), CD4-Cy5 (eBioscience, 15–0049), and CD8-Cy5 (eBioscience, 15–0088) were added to the appropriate tubes containing PBMCs and incubated in the dark at 4°C for 30 minutes. Cell suspension was centrifuged three times at 800 x g for 5 minutes to remove excess staining and then suspended in 1 ml 1X cold PBS. The fluorescence was quantified by flow cytometry. Results were analyzed using FlowJo software version 7.6.5.

### Malondialdehyde assay

MDA is a byproduct of lipid peroxidation. Once MDA forms an adduct with thiobarbituric acid at 90°C -100°C, a color change occurs which can be measured colorimetrically at 530–540 nm. MDA levels in plasma samples, monocyte and RBC lysates from healthy subjects and individuals with T2DM were measured using a TBARS Assay Kit (Cayman Chemical, 10009055).

### Isolation of monocytes

PBMCs isolated from the peripheral blood of healthy subjects and individuals with T2DM were washed 3 times with 1X PBS, re-suspended in RPMI with L-glutamine, HEPES (Corning Cellgro, 1–041-CM) and 5% human AB serum, plated on a 96 well tissue culture plate pre-coated with 0.005% poly-L-lysine, and incubated overnight at 37°C to allow monocyte adherence.

### *In vitro* differentiation of macrophages

Following overnight incubation, the non-adherent cells were removed from the plate. Monocytes that remain adhered to the plate were replenished with fresh RPMI media, and incubated for 7 days at 37°C to allow differentiation to macrophages (HMDM).

### Macrophage infection studies

We tested the effects of GSH-enhancing agents in improving the ability of isolated macrophages from T2DM to control *M*. *tb* infection. HMDM from healthy subjects and individuals with T2DM were brought into a Biosafety Level-3 facility (BSL-3) for infection with H37Rv, a laboratory strain of *M*. *tb* with multiplicity of infection of 10:1. The infected macrophages were incubated for 2 hours to allow phagocytosis of bacteria. Unphagocytosed bacteria were removed by washing the infected macrophage cultures three times with 1X PBS. Infected macrophages were maintained for 5 days in fresh RPMI with and without additives such as N-acetyl cysteine (NAC, 10 mM and 20 mM) and liposomal glutathione (***l***GSH, 10 μM and 20 μM ***l***GSH). NAC is a GSH precursor, whereas ***l***GSH is a formulation containing reduced GSH encapsulated in liposomes and can be readily ingested by macrophages. ***l***GSH was generously provided by Dr. Frederick Guilford, Your Energy Systems, CA. Infected HMDM were terminated at 1 hour and 5 days post-infection. Infected macrophages were lysed by adding 200 μl of cold sterile water. Lysates were collected and plated on 7H11 containing glycerol and albumin dextrose complex (ADC).

### Assay of cytokines in plasma samples from healthy subjects and individuals with T2DM

The Cytokine profile in the plasma samples isolated from individuals with T2DM was compared to healthy volunteers. Cytokine levels in plasma samples were determined by sandwich enzyme linked immunosorbent assay (ELISA) using assay kits from eBioscience (ELISA Ready-Set-Go: IFN-γ cat # 88–7316, IL-1 cat # 88–7010, IL-2 cat # 88–7025, IL-6 cat # 88–7066, IL-10 cat # 88–7106, IL-12 cat # 88–7126, TGF-β cat # 88–8350, TNF-α cat # 88–7346).

### Statistical analysis

Statistical data analysis was performed using Graph Pad Prism Software version 6. Levels of total GSH, rGSH, GSSG, GSH synthesis and metabolism enzymes, ROX, MDA, and cytokines were compared between individuals with T2DM and healthy volunteers using the unpaired t-test with Welch correction. Reported values are in means ± standard error, p<0.05 was considered significant (*p<0.05, **p<0.005, and ***p<0.0005).

## Results

### Assay of GSH levels in plasma, RBCs and monocytes from healthy subjects and individuals with T2DM

**Fig 1:**
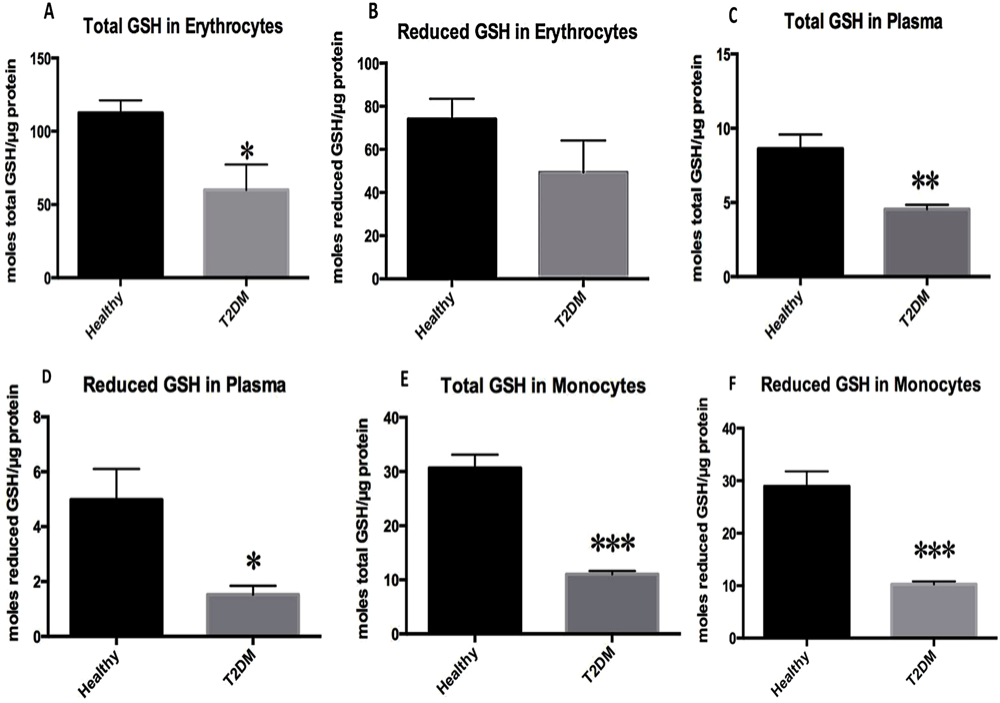
Assay of total and rGSH in RBCs, plasma, and monocytes from healthy subjects and individuals with T2DM. RBCs, plasma, and monocytes were isolated by density centrifugation from peripheral blood drawn from healthy volunteers and individuals with T2DM. GSH assay was performed using a colorimetric assay kit from Arbor Assays. Total GSH in RBCs isolated from individuals with T2DM was significantly lower compared to the healthy individuals (Fig. 1A). There was an observable decrease in rGSH in RBCs isolated from individuals with T2DM compared to healthy volunteers (Fig. 1B). Total GSH in plasma samples from individuals with T2DM was significantly lower compared to the healthy individuals (Fig. 1C). There was a significant decrease in rGSH in plasma samples from individuals with T2DM compared to healthy volunteers (Fig. 1D). Total GSH in monocyte samples isolated from individuals with T2DM was significantly lower compared to total GSH measured in monocytes from healthy individuals (Fig. 1E). There was a significant decrease in rGSH in monocyte samples isolated from individuals with T2DM compared to healthy volunteers (Fig. 1F). Data represent means ±SE from 10 healthy individuals and 10 individuals with T2DM.

Assay of GSH levels in RBC samples showed that the levels of both total GSH and rGSH were significantly lower in individuals with T2DM compared to healthy volunteers (Fig. 1A and 1B). In comparison to healthy subjects, there was a two-fold decrease in the levels of total GSH in RBCs isolated from individuals with T2DM (Fig. 1A). Assay of GSH in plasma samples from T2DM showed a similar trend, showing a significant decrease (two-fold decrease) in the levels of both total GSH and rGSH in plasma samples from individuals with T2DM compared to healthy subjects (Fig. 1C and 1D). Consistent with our observations in RBCs and plasma, we observed a significant three-fold decrease in the levels of GSH in monocytes isolated from individuals with T2DM compared to healthy volunteers (Fig. 1E and 1F).

### Quantifying the levels of enzymes involved in the synthesis and metabolism of GSH

**Fig 2:**
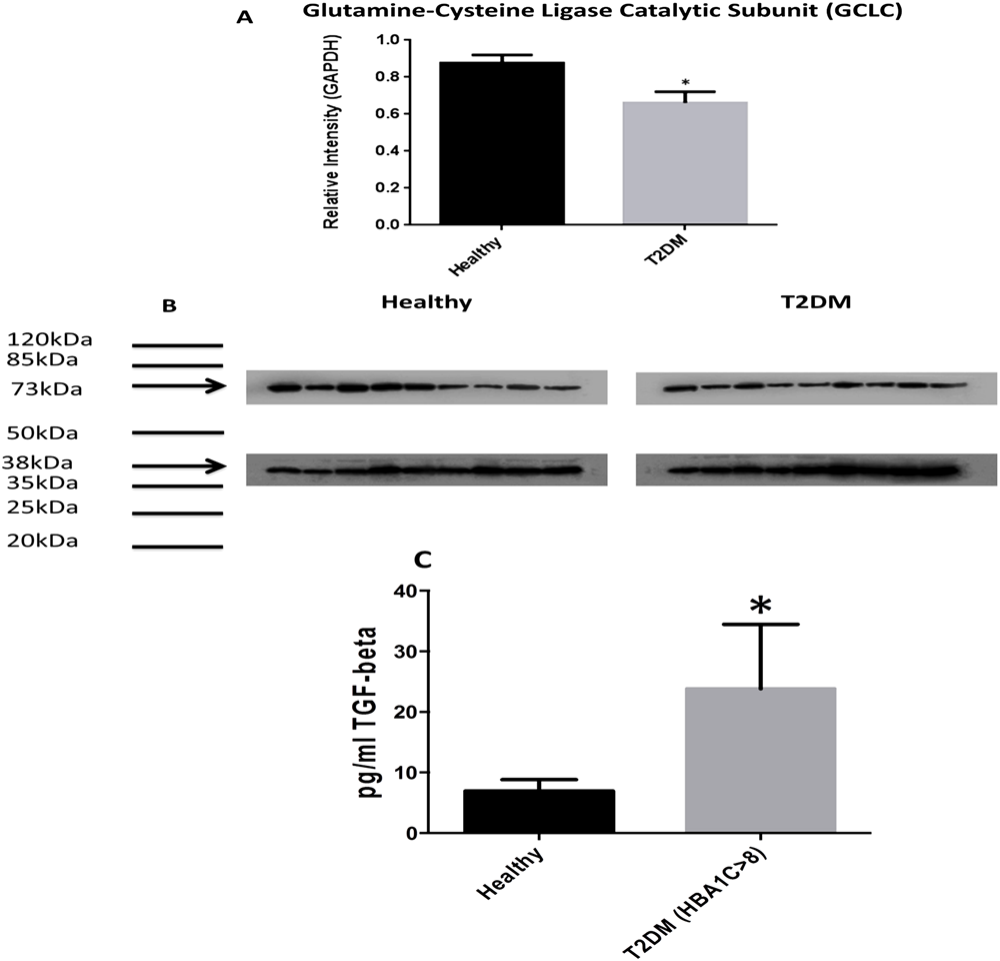
Assay of TGF-β levels in plasma and western blot analysis of a GSH *de novo* synthesis enzyme, GCLC, in RBCs from healthy subjects and individuals with T2DM. Western blot results were corrected for GAPDH and reported as relative intensity compared to GAPDH (Fig. 2A). Results show significantly lower levels of GCLC measured in RBCs isolated from individuals with T2DM compared to healthy subejcts (A; n = 9 healthy, n = 9 T2DM) and B; n = 9 healthy, n = 9 T2DM). Fig. 2B illustrates western blot images of GCLC and GAPDH in RBCs isolated from healthy subjects and individuals with T2DM. Assay of TGF-β was performed using an ELISA Ready-Set-Go kit from eBioscience (Fig. 2C). There was a significant increase in TGF-β production in plasma samples isolated from individuals with T2DM with HbA1c>8 compared to healthy volunteers (n = 5).

**Fig 3:**
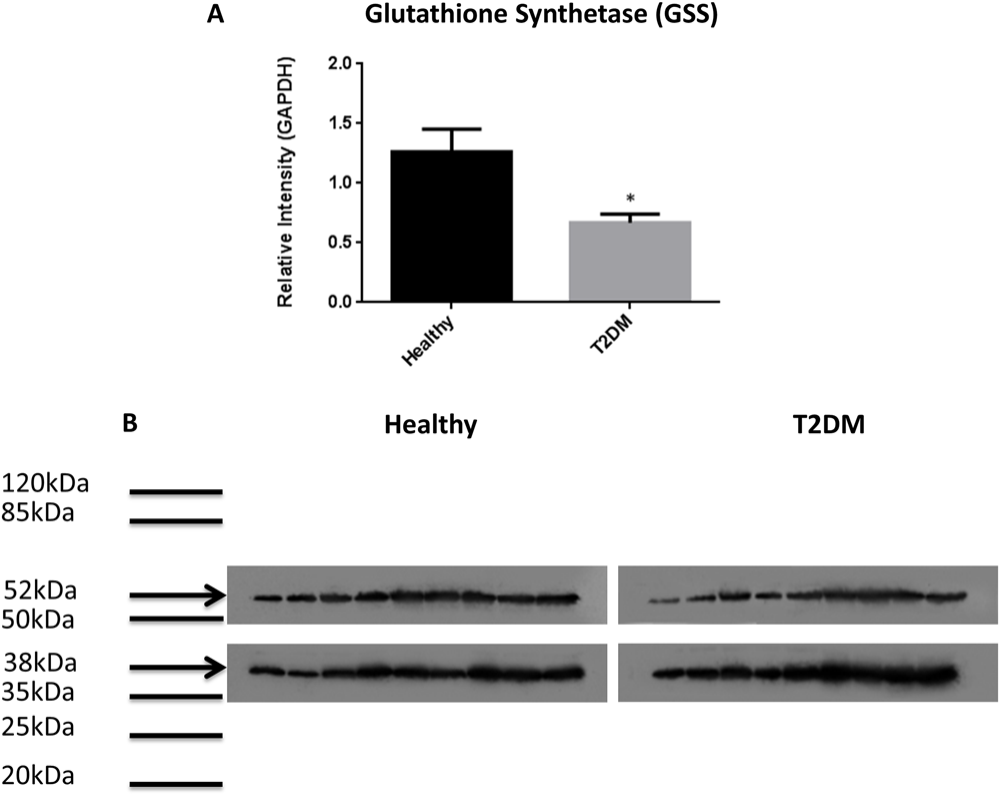
Western blot analysis of a GSH *de novo* synthesis enzyme, GSS, in RBCs of individuals with T2DM compared to healthy subjects. Western blot results were corrected for GAPDH and reported as relative intensity compared to GAPDH (Fig. 3A). Significant decrease in the levels of GSS was observed in RBCs isolated from individuals with T2DM compared to healthy volunteers. (A; n = 9 healthy, n = 9 T2DM and B; n = 9 healthy, n = 9 T2DM). Fig. 3B illustrates western blot images of GSS and GAPDH in RBCs isolated from healthy subjects and individuals with T2DM.

**Fig 4:**
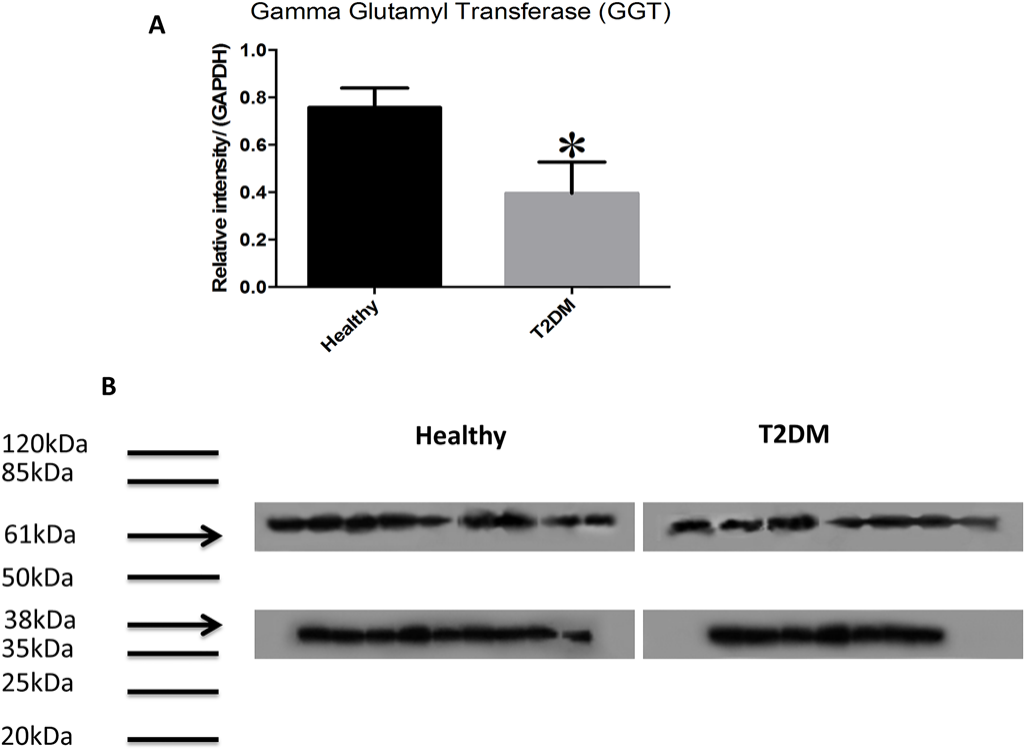
Western blot analysis of a GSH enzyme, GGT, in RBCs from healthy subjects and individuals with T2DM. Western blot results were corrected for GAPDH and reported as relative intensity compared to GAPDH (Fig. 4A). In comparison to healthy subjects, we observed significantly lower levels of GGT in RBCs isolated from individuals with T2DM (A; n = 9 healthy, n = 7 T2DM and B; n = 9 healthy, n = 7 T2DM). Fig. 4B illustrates western blot images of GGT and GAPDH in RBCs isolated from healthy subjects and individuals with T2DM.

**Fig 5:**
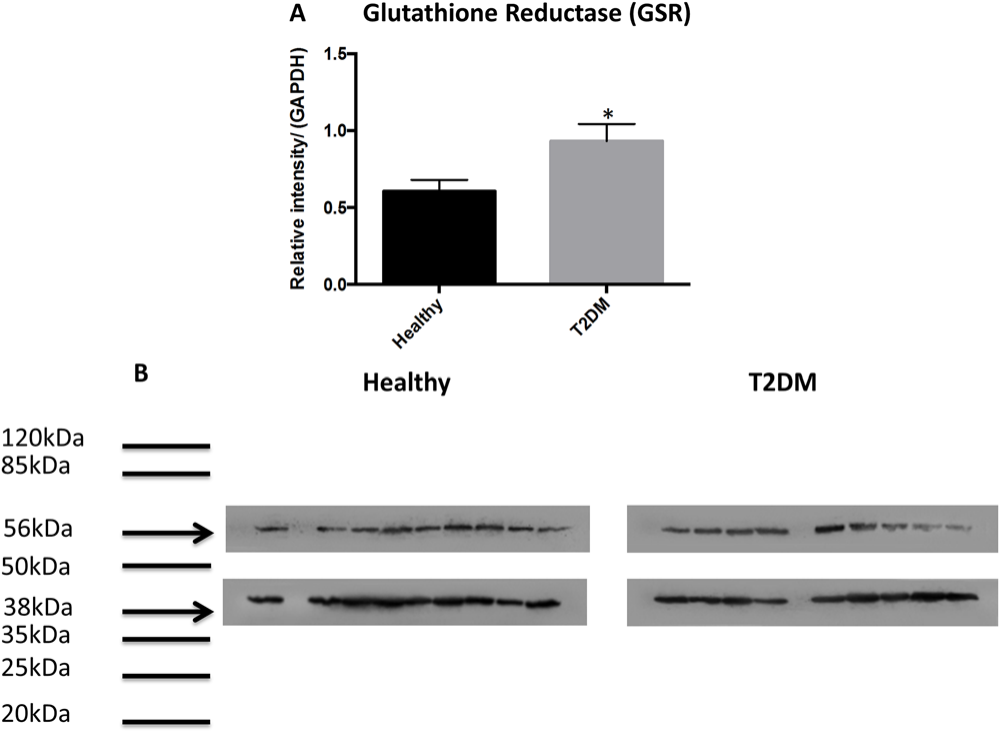
Western blot analysis of a GSH recycling enzyme, GSR, in RBCs from healthy subjects and individuals with T2DM. Western blot results were corrected for GAPDH and are reported as relative intensity compared to GAPDH (Fig. 5A). In comparison to healthy subjects, we observed a significant increase in the levels of GSR in RBCs from individuals with T2DM (A; n = 9 healthy, n = 9 T2DM and B; n = 9 healthy, n = 9 T2DM). Fig. 5B illustrates western blot images of GGT and GAPDH in RBCs isolated from healthy subjects and individuals with T2DM.

Western blot analysis showed significantly lower levels of GCLC (catalytic unit of the rate limiting step enzyme involved in the synthesis of GSH) in RBCs isolated from individuals with T2DM compared to healthy subjects (Fig. 2A, B). Determination of TGF-β levels in the plasma samples isolated from healthy subjects and individuals with T2DM showed that decreased expression of GCLC correlated with significant increase in the levels of TGF-β in plasma samples from individuals with T2DM (Fig. 2C). There was 50% reduction in the expressions of GSS and GGT levels in RBCs isolated from individuals with T2DM compared to healthy subjects (Fig. 3, 4). In contrast, GSR levels were significantly higher in RBCs isolated from individuals with T2DM compared to healthy volunteers (Fig. 5).

### Measurement of oxidative stress in T2DM

**Fig 6:**
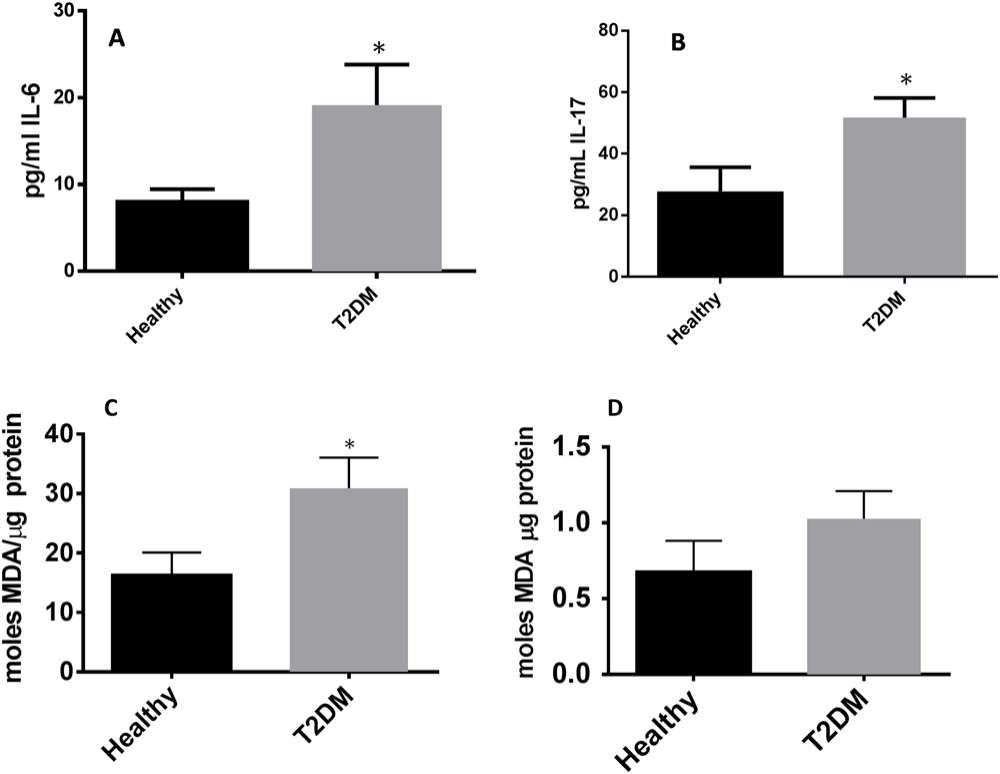
Assay of IL-6 and IL-17 in healthy subjects and individuals with T2DM (Fig. 6A and B) and Assay of MDA in healthy subjects and individuals with T2DM (Fig. 6C, D, and E). Assay of IL-6 (Fig. 6A) and IL-17 (Fig. 6B) were performed using an ELISA Ready-Set-Go kit from eBioscience. IL-6 and IL-17 levels were significantly higher in plasma samples from individuals with T2DM. Results on IL-6 and IL-17 represent means ±SE from 10 healthy individuals and 10 individuals with T2DM. MDA assay was performed using a TBARS kit from Cayman Chemical. There was a statistically significant and two-fold increase in the levels of MDA in plasma samples from individuals with T2DM compared to healthy volunteers (Fig. 6C). There was an observable increase in the levels of MDA in monocytes isolated from individuals with T2DM compared to healthy volunteers (Fig. 6D). MDA levels were also assayed in RBCs isolated from individuals with T2DM and healthy subjects. Individuals with T2DM with HbA1c>8 had significantly higher levels of MDA compared to healthy individuals (Fig. 6E).

**Fig 7:**
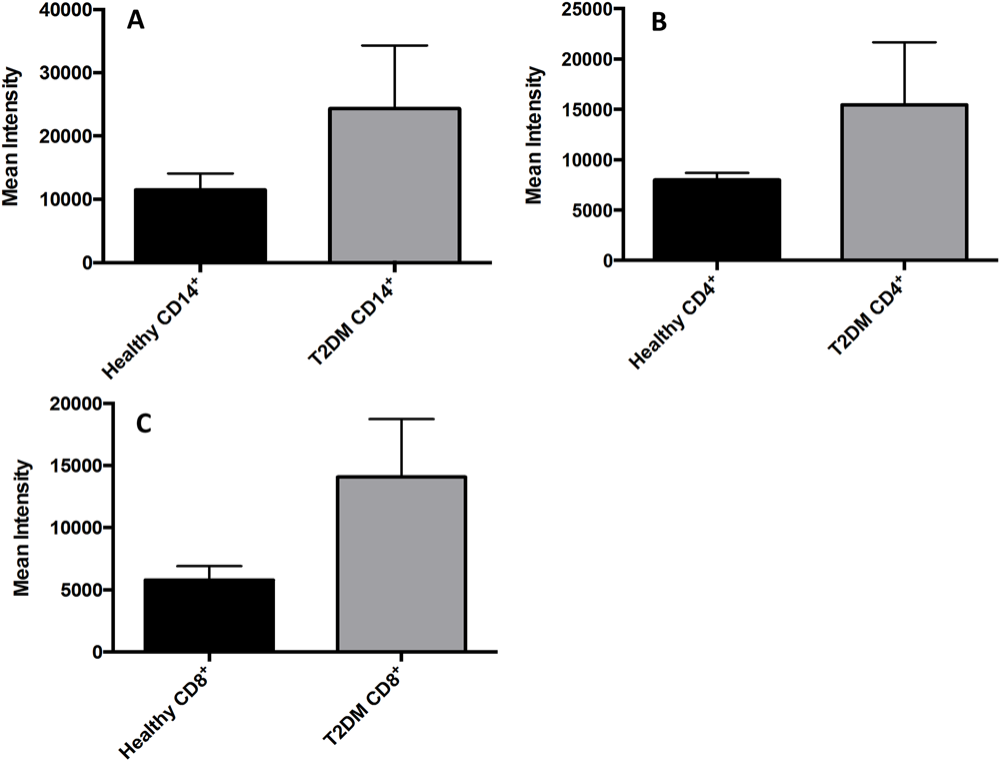
Measurement of ROS in CD14^+^ cells, CD4^+^ T-cells, and CD8^+^ T-cells by cellROX stain mean intensity in T2DM patients compared to healthy. CD14^+^cells were stained with cellROX green reagent, a marker of ROS, and a CD14 cell marker, CD14-PE. CD14^+^-ROX^+^ cells’ mean intensity was analyzed by FLOW cytometry. There was an observable increase in ROX mean intensity in CD14^+^ cells isolated from individuals with T2DM compared to healthy volunteers (Fig. 7A). CD4^+^cells were also stained with cellROX green reagent and a CD4 cell marker, CD4-Cy5. CD4^+^-ROX^+^ cells’ mean intensity was analyzed by FLOW cytometry. There was an observable increase in ROX mean intensity in CD4^+^ T-cells isolated from individuals with T2DM compared to healthy volunteers (Fig. 7B). CD8^+^cells were stained with cellROX green reagent and a CD8 cell marker, CD8-Cy5. CD8^+^-ROX^+^ cells’ mean intensity was analyzed by FLOW cytometry. There was an observable increase in ROX mean intensity in CD8^+^ T-cells isolated from individuals with T2DM compared to healthy volunteers (Fig. 7C). Data represents mean ±SE from 5 healthy individuals and 5 individuals with T2DM.

**Fig 8:**
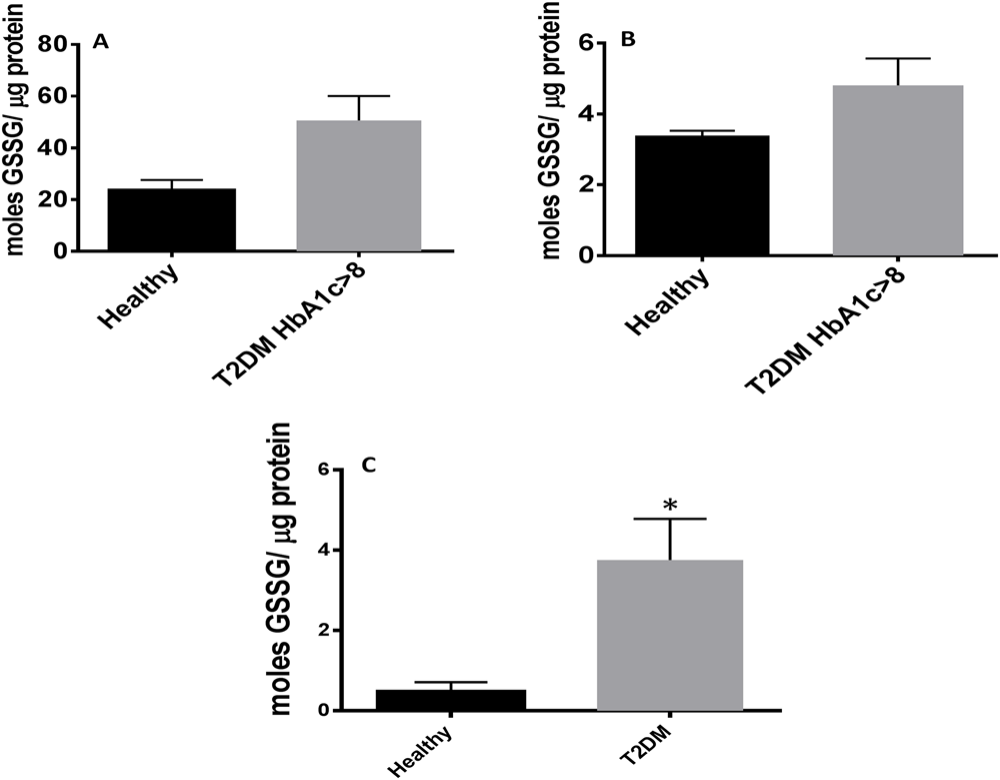
Assay of GSSG in RBCs, plasma, and monocytes isolated from healthy subjects and individuals with T2DM. GSSG was measured using a GSH colorimetric assay from Arbor Assays. We observed two-fold increase in levels of GSSG in RBCs isolated from individuals with T2DM with HbA1c>8 compared to healthy volunteers (Fig. 8A). There was an observable increase in levels of GSSG in plasma samples isolated from individuals with T2DM with HbA1c>8 compared to healthy volunteers (Fig. 8B). We also observed a significant eight-fold increase in the levels of GSSG in monocytes isolated from individuals with T2DM compared to healthy volunteers (Fig. 8C). Data represent means ±SE from 10 healthy individuals and 10 individuals with T2DM.

Levels of MDA in RBCs isolated from individuals with T2DM were compared to healthy volunteers. Further breakdown of the T2DM group by HbA1c levels showed that MDA in individuals with HbA1c more than eight percent (8%) was significantly higher compared to healthy individuals. Although an observable increase in the levels of MDA was observed in RBCs from T2DM with HbA1c less than eight percent (8%), the increase was not statistically significant (Fig. 6C). We also observed a two-fold increase in the levels of MDA in plasma and monocytes of T2DM compared to healthy volunteers (Fig. 6D and 6E). Flow cytometry analysis of cellROX green reagent, a marker of ROS, revealed that cellROX mean intensity was distinctly higher in CD14^+^ cells, CD4^+^ T-cells, and CD8^+^ T-cells from individuals with T2DM compared to healthy volunteers (Fig. 7A-C). Assay of GSSG showed a marked increase in the levels of GSSG in RBCs isolated from individuals with T2DM with HbA1c more than eight percent (8%) compared to healthy volunteers (Fig. 8A). We also observed an increased in levels of GSSG in the plasma samples from individuals with T2DM with HbA1c more than eight percent 8% compared to healthy volunteers (Fig. 8B). Finally, we observed a significant increase in the levels of GSSG in monocytes isolated from T2DM subjects compared to healthy subjects (Fig. 8C). In summary, there was an observed increase in GSSG and MDA levels in RBCs, plasma, and monocytes from individuals in the T2DM group. Similarly, cellROX mean intensity was higher in CD14^+^ cells, CD4^+^ T-cells, and CD8^+^ T-cells from individuals with T2DM compared to healthy volunteers.

### Intracellular survival of H37Rv inside HMDM from healthy subjects and individuals with T2DM

**Fig 9:**
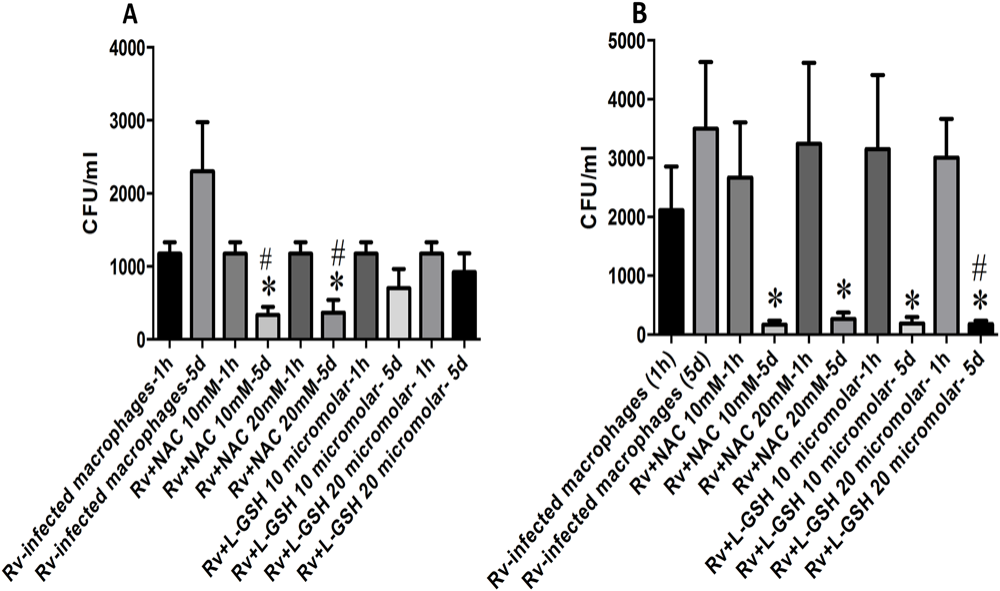
Intracellular survival of H37Rv in macrophages from healthy volunteers and individuals with T2DM. Macrophages were infected with H37Rv, a laboratory strain of *M*.*tb*, with multiplicity of infection 10:1. One hour post-infection, infected macrophageswere treated with 10 mM and 20 mM NAC and 10 μM and 20 μM *l*GSH. Infected macrophages were terminated at 1h and 5 days post-infection. Lysates were plated on 7H11 medium with ADC to determine *M*.*tb* growth. Data represent means ±SE from 5 healthy individuals (Fig. 9A) and 5 individuals with T2DM (Fig. 9B). Experiments were performed in triplicates. *p<0.05 when comparing H37Rv-infected macrophages 5 day time point results to any other 5 day time point categories while #p<0.05 when comparing 1 hour and 5 day time points of the same treatment category.

We observed a two-fold increase in the intracellular survival of H37Rv in macrophages from healthy subjects. Treatment of HMDM from healthy subjects and individuals with T2DM with NAC (10 mM and 20 mM) resulted in significant decrease in the intracellular viability of H37Rv (Fig. 9A and B). ***l***GSH treatment (10 μM and 20 μM) of HMDM isolated from individuals with T2DM resulted in significant inhibition in the intracellular growth of H37Rv. The concentration of ***l***GSH used in our studies is 1000-fold lower compared to NAC. L-GSH is more efficacious in controlling *M*. *tb* infection in macrophages from T2DM (Fig. 9B) compared to healthy subjects because of its superior restorative effects since individuals with T2DM express low levels of enzymes involved in *de novo* synthesis of GSH. Our findings confirm that enhancing the levels of GSH in HMDM from healthy and T2DM result in improved control of *M*. *tb* infection.

### Assay of cytokines in plasma samples from healthy subjects and individuals with T2DM

**Fig 10:**
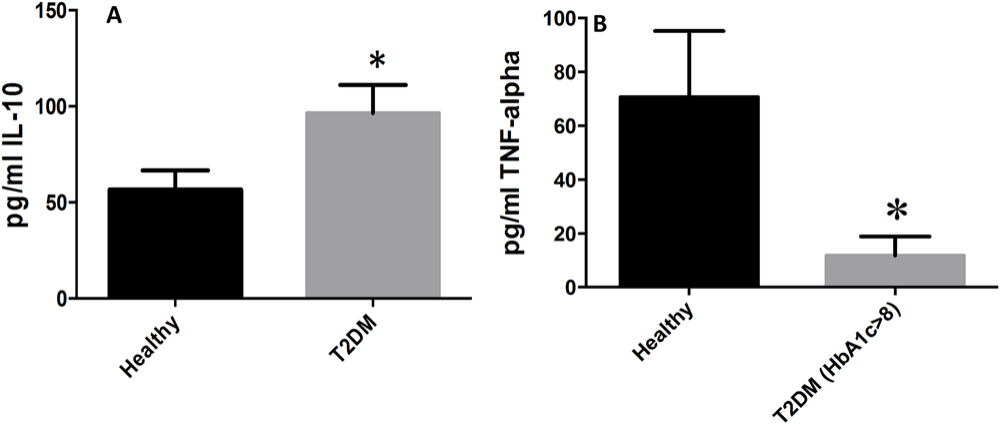
Assay of IL-10 and TNF-α in the plasma samples from healthy subjects and individuals with T2DM. Assay of IL-10 and TNF-α, in plasma was performed using an ELISA Ready-Set-Go kit from eBioscience. Data represent means ±SE, *p<0.05. There was two-fold increase in IL-10 (Fig. 10A) in plasma samples isolated from individuals with T2DM compared to healthy volunteers (n = 10 healthy, n = 10 T2DM). TNF-α was significantly lower in plasma samples isolated from individuals with T2DM and HbA1c>8 compared to healthy volunteers (Fig. 10B n = 10 healthy, n = 5 T2DM).

**Fig 11:**
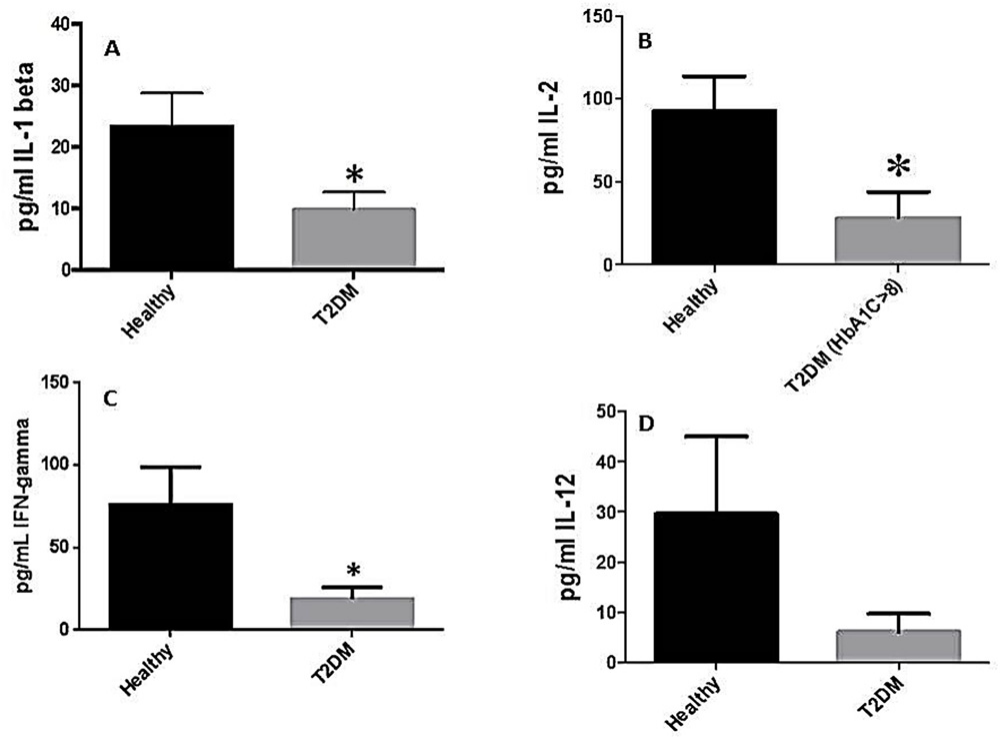
Assay of IL-1β, IL-2, IFN-γ, and IL-12 levels, in plasma samples from healthy subjects and individuals with T2DM. Plasma was isolated from peripheral blood of healthy volunteers and individuals with T2DM. Assay of IL-1β (Fig. 11A), IL-2 (Fig. 11B), IFN-γ (Fig. 11C), and IL-12 (Fig. 11D) levels, in plasma was performed using an ELISA Ready-Set-Go kit from eBioscience. Data represent means ±SE from 10 healthy individuals and 10 individuals with T2DM except panel B in which the sample size for individuals with T2DM and HbA1c>8 is n = 5.

Assay of different cytokines in plasma samples from healthy subjects and individuals with T2DM was performed using a sandwich ELISA. The pro-inflammatory cytokines IL-6 and IL-17 were significantly elevated in plasma samples isolated from T2DM patients compared to healthy volunteers (Fig. 6A, 6B). Increased levels of IL-6 and IL-17 in plasma samples from individuals with T2DM correlated with enhanced oxidative stress (Fig. 6C, 6D, 6E, 7A, 7B, 7C, 8A, 8B and 8C). We observed a significant and two-fold increase in the levels of IL-10, an immunosuppressive cytokine in the plasma samples from individuals with T2DM (Fig. 10A). On the other hand, there was a five-fold decrease in the levels of TNF-α (implicated in the formation of granuloma and activating the macrophages) in plasma samples from individuals with T2DM with HbA1c>8 (Fig. 10B). We also observed a twofold decrease in the levels of IL-1β and IFN-γ in plasma samples isolated from individuals with T2DM compared to healthy volunteers (Fig. 11A and 11C). Similarly, there was a significant decrease in the levels of IL-2 (Fig. 11B), and a six-fold decrease in the levels of IL-12 (Fig. 11D), in plasma samples isolated from individuals with T2DM. In summary, there was an increase in the immunosuppressive cytokine, IL-10 while there was a notable decrease in the levels of protective cytokines, TNF- α, IL-1β, IL-2, IL-12, and IFN-γ in individuals with T2DM (Fig. 11A-D).

## Discussion

Total GSH is comprised of rGSH and GSSG. It is the rGSH form that exhibits antioxidant activity. In the presence of ROS, this form interconverts into GSSG, which lacks antioxidant activity. At initial measurements, we observed that the levels of total GSH and rGSH were compromised in RBCs, plasma, and monocytes derived from individuals with T2DM (Fig. 1).

GCL, a rate-limiting step enzyme involved in the *de novo* synthesis of GSH, links glutamine and cysteine to form γ-glutamylcysteine. Another enzyme, GSS, then links γ-glutamylcysteine to glycine to form GSH. It has been reported that TGF-β can downregulate the expression of GCLC [14]. Western blot analysis of GSH synthetic-enzymes showed that GCLC (Fig. 2A) and GSS (Fig. 3) protein levels were significantly decreased in RBCs derived from individuals with T2DM compared to healthy subjects. We also observed a significant increase in the levels of TGF-β (Fig. 2B) in the plasma samples from T2DM volunteers compared to healthy individuals indicating a possible underlying mechanism that is responsible for decreasing the expression of GCLC in individuals with T2DM.

Orlowski and Meister described GGT as an amino acid transport system in the γ-glutamyl cycle [16,18]. Forman *et*.*al* (2009) reported that due to the inability of GSH to cross the plasma membrane, it must be completely hydrolyzed and resynthesized after uptake [18]. GGT fulfills this role by cleaving the γ-glutamyl moiety of GSH and releasing cysteine, an essential amino acid required to resynthesize GSH. Thus, in this manner, GGT is responsible for the breakdown and subsequent translocation of GSH into the intracellular space. GGT protein levels were significantly lower in RBCs isolated from individuals with T2DM compared to healthy volunteers (Fig. 4). This finding along with reduced levels of GCLC and GSS explain the cause for diminished levels of total GSH and rGSH in individuals with T2DM.

Interestingly, there was a significant increase in the levels of GSR in RBCs from individuals with T2DM compared to healthy subjects (Fig. 5). Our results imply that despite significant increase in the levels of GSR, there will not be efficient conversion of GSSG to rGSH in individuals with T2DM. This is due to insufficient levels of NADPH, since most of the NADPH will be rapidly utilized by aldose reductase (AR) for the polyol pathway [11]. Our findings unveil the process leading to GSH deficiency in individuals with T2DM.

The increased levels of free radicals in individuals with T2DM can be attributed to enhanced production of pro-inflammatory cytokines such as IL-6 and IL-17, the combined effects of these cytokines can result in excessive formation of free radicals. IL-6, a cytokine produced by monocytes, B cells, neutrophils, and T cells has been shown to be elevated in individuals with T2DM [20]. High levels of IL-6 in adipose tissue of people who are obese have been associated with lower insulin mediated glucose uptake. Furthermore, IL-6 increased ROS production in proximal convoluted tubules (PCT), leading to increased glucose reabsorption activities of sodium glucose linked transporter (SGLT) [21]. Overall, higher IL-6 production leads to increased formation of free radicals, lower insulin mediated glucose uptake, and increased glucose reabsorption [19–21]. Increased levels of pro-inflammatory cytokines lead to an excessive increase in free radicals and increased scavenging activity of GSH.

The increased levels of free radicals in individuals with T2DM can also be attributed to the polyol pathway. After conversion of glucose to sorbitol by AR, sorbitol is converted to fructose. This second step in the polyol pathway converts NAD^+^ to NADH leading to production of NADH oxidase (NOx) and increase in ROS [11,19]. The polyol pathway also results in the production of advanced glycation end products (AGE) that bind to receptors for advanced glycation end products (RAGE) leading to the generation of ROS [11]. With continuous production of pro-inflammatory cytokines and increased free radical formation, GSH becomes depleted faster than it can be synthesized or recycled.

We observed a significant increase in the levels of IL-6 and IL-17 in the plasma samples isolated from individuals with T2DM (Fig. 6A and B). MDA, a byproduct of lipid peroxidation, forms adduct with thiobarbituric acid that can be measured spectrophotometrically. We observed a significant increase in the levels of MDA in RBCs, plasma, and monocytes from T2DM (Fig. 6C, D and E). Similarly, ROS production in CD14^+^ cells, CD4^+^ T-cells, and CD8^+^ T-cells were greater in individuals with T2DM compared to healthy volunteers (Fig. 7A, B and C). Increased MDA levels in RBCs, plasma and monocytes from T2DM was accompanied by increased levels of GSSG (Fig. 8), further confirming that there is systemic oxidative stress in individuals with T2DM.

Our lab has previously reported that GSH has direct antimycobacterial effects. We demonstrated that the virulent laboratory strain of *M*. *tb*, H37Rv, is sensitive to physiological concentrations of GSH [12, 22–26]. We also showed that treatment of macrophages with 10mM NAC (a GSH precursor) resulted in control of intracellular *M*. *tb* infection. Treatment of *M*. *tb* infected macrophages with liposomal GSH (*l*GSH) added at 10 μM and 20 μM concentrations, also decreased the growth *of M*. *tb* inside the macrophages derived from individuals with HIV infection [27]. These results confirm the direct antimycobacterial effects of GSH.

In this study, we observed an increase in the intracellular growth of *M*. *tb* inside HMDM from healthy subjects and individuals with T2DM between the initial and final time point of termination (Fig. 9A and B). Treatment of HMDM from healthy subjects with NAC added at millimolar concentrations (10 mM and 20 mM) led to significant reduction in the intracellular growth of *M*. *tb*. Treatment of HMDM from healthy subjects with micromolar concentrations of ***l***GSH (1000 X lower compared to NAC-treatment) resulted in effective inhibition in the growth of H37Rv. However, the inhibition in the growth of H37Rv was not statistically significant. In contrast to healthy subjects, treatment of HMDM derived from individuals with T2DM with micromolar concentrations ***l***GSH resulted in a statistically significant decrease in the growth of H37Rv. Since the *de novo* synthesis enzymes required for the synthesis of GSH are compromised in T2DM, supplying macrophages with a whole molecule of GSH in a liposomal formulation was very effective in reducing the growth of *M*. *tb* in T2DM, despite adding a 1000-fold lower concentration (Fig. 9B). Since healthy subjects have normal levels of GSH *de novo* synthetic enzymes, NAC treatment alone was sufficient to reduce the intracellular survival of *M*. *tb* inside the macrophages (Fig. 9A).

Cytokines are vital in eliciting innate and adaptive immune functions against infection [25]. It is our prediction that individuals with T2DM will have reduced levels of protective cytokines such as TNF-α, IL-1β, IL-2, IL-12 and IFN-γ, and increased levels of IL-10, an immunosuppressive cytokine thereby leading to their increased susceptibility to *M*. *tb* infection. We observed decreased levels of TNF-α in plasma samples from individuals with T2DM (Fig. 10B), along with increased levels of IL-10 (Fig. 10A). TNF-α is vital for the formation of granuloma during *M*. *tb* infection. Compromised levels of TNF-α will impair the formation of granuloma leading to active TB. IL-10, an immunosuppressive cytokine, inhibits the effector mechanisms inside macrophages [28, 29]. Results of our studies indicate that low levels of TNF-α is accompanied by an increase in IL-10, an anti-inflammatory cytokine responsible for mitigating TNF-α (Fig. 10A and B) [26, 28]. These results demonstrate that the susceptibility of the T2DM group to *M*. *tb* infection can be attributed to failed granuloma formation.

We also observed that the levels of IL-1β and IFN-γ are compromised in plasma samples from individuals with T2DM (Fig. 11A and C). Decrease in IL-1β may lead to the decreased expression of inducible nitric oxide synthase, the enzyme responsible for nitric oxide (NO) synthesis [30–32]. On the other hand, IFN-γ stimulates the macrophages to release NO [30]. More importantly, NO can be coupled with GSH to form S-nitrosoglutathione (GSNO) [22]. We previously reported that GSNO has mycobactericidal effects against *M*. *tb* at physiological concentrations [22]. Both IL-1β and IFN-γ are important for inducing NO production, which serves as an effector molecule involved in the killing of *M*. *tb* [30]. Decreased levels of both these cytokines lead to increased susceptibility to *M*. *tb* [33].

Importantly, we demonstrated that the levels of IL-2 and IL-12 were also diminished in plasma samples isolated from individuals with T2DM (Fig. 11B and D). IL-2 is important in maintaining the viability of CD4^+^ and CD8^+^ T-cells. Decreased levels of IL-2 can reduce the number of effector T-cells. More specifically, a decrease in CD4^+^ T-cell induced IL-2 production can lead to the failed activation and differentiation of CD8^+^ T-cells [34, 35]. IL-12 is a polarizing cytokine which signals the differentiation of naïve CD4^+^ cell into the T_H_1 subset [36–38]. GSH has been reported to increase IL-12 induction in active *M*. *tb* infection [12,37,38]. This increase in IL-12 urges CD4^+^ cells to select a T_H_1 identity over a T_H_2 identity [38]. The T_H_1 subset produces IFN-γ, which leads to the release of NO, an effective defense against *M*. *tb* [9, 36]. Thus, an increase in IL-12 leads to an increase in IFN-γ. Therefore, decreased levels of IL-12 observed in individuals with T2DM (Fig. 11D) ultimately leads to less activation of macrophages due to decreased levels of T_H_1 cells. Compromised levels of IL-2 and IL-12 in individuals with T2DM will diminish effector T-cell activities against *M*. *tb*

Overall, decreased levels of TNF-α, IL-1β, IFN-γ, IL-2, and IL-12 in plasma from T2DM individuals will lead to the failed formation of the granuloma, decreased NO release, diminished activation of macrophages, and weakened T-cell activity. These factors, along with an increase in the immunosuppressive cytokine IL-10, result in individuals with T2DM becoming increasingly susceptible *M*. *tb* infection.

In summary, the total and reduced forms of GSH were significantly compromised in RBCs, plasma, and monocytes isolated from individuals with T2DM. Decreased levels of GSH in individuals with T2DM were accompanied by diminished expressions of GSH synthetic enzymes such as GCLC, GSS, and GGT. Furthermore, we observed a significant increase in the levels TGF-β and GSR enzyme in individuals with T2DM. Our findings also indicate that an increase in IL-6 and IL-17 production leads to an increase in ROS and glucose reuptake. In addition, the polyol pathway induces further ROS production by converting excessive glucose to fructose. These factors, along with diminished synthesis of GSH *de novo* and metabolic enzymes, result in the depletion of rGSH. Importantly, restoring the levels of GSH in monocytes from individuals with T2DM resulted in improved innate immune responses against *M*. *tb* infection.
